# Maize ZmBES1/BZR1-7 Transcription Factor Positively Regulates Drought Tolerance in Transgenic *Arabidopsis* and Rice

**DOI:** 10.3390/plants15152359

**Published:** 2026-07-31

**Authors:** Huaming Duan, Xin Zhang, Yiran Zhao, Ruxiu He, Liu He, Didi Lina, Tao Wan, Yajie Liu, Qingqing Yang, Fengling Fu, Haoqiang Yu

**Affiliations:** Maize Research Institute, Sichuan Agricultural University, Chengdu 611130, China

**Keywords:** maize, transcription factor, BES1/BZR1, drought, ectopic expression

## Abstract

Drought stress significantly limits agricultural productivity and threatens food security. The identification of drought tolerance genes and the dissection of their regulatory networks in crops are crucial for ensuring future food production. The BRI1-EMS1 suppressor (BES1)/brassinazole-resistant 1 (BZR1) family is a kind of plant-specific transcription factor that regulates growth, development, and stress responses in plants. Here, the maize *ZmBES1/BZR1-7* gene was cloned and transformed into *Arabidopsis* and rice to characterize plant phenotypes under drought stress conditions. Sequence analysis revealed that the ZmBES1/BZR1-7 protein contains a conserved basic helix-loop-helix (bHLH) domain, localizes to the nucleus, and exhibits transcriptional activation activity. Expression profiling revealed that *ZmBES1/BZR1-7* is induced by drought stress in maize. Overexpression of *ZmBES1/BZR1-7* significantly enhanced drought tolerance in both transgenic *Arabidopsis* and rice. Under drought stress conditions, all transgenic lines showed higher survival rates and relative water content (RWC), increased root length, reduced relative electrolyte leakage (REL) and malondialdehyde (MDA) content, as well as higher yield of transgenic rice than the wild type. Integrated analyses of RNA-seq and qRT-PCR assays demonstrated that ZmBES1/BZR1-7 modulates transcription of stress-responsive genes to enhance drought tolerance. Collectively, the study provides insights into the molecular mechanisms by which ZmBES1/BZR1-7 mediated drought stress response in maize.

## 1. Introduction

Drought, high salinity, and high temperature are major abiotic stresses that severely limit agricultural productivity and threaten food security [1]. Climate warming, population surge, and increasing water scarcity will intensify land aridification and salinization in the coming decades [2,3]. In response to environmental stresses, plants have evolved diverse adaptive regulatory cascades, including physiological, biochemical, and molecular changes [4,5,6,7]. Gene transcriptional regulation plays a critical role in the plant response to abiotic stress, which is modulated by transcription factors (TFs) regulating the expression of nuclear genes by binding to their promoter regions [8]. It has been reported that TFs, including NAC, WRKY, MYB, BES1/BZR1, and bZIP, are involved in plant resistance to abiotic stress [9,10,11,12].

As plant-specific TFs, BES1/BZR1s integrate multiple regulatory networks and bind to E-box or BRRE motifs located in the promoters to modulate their expression, consequently governing plant physiological and biochemical responses [13,14,15]. The biological functions of BES1/BZR1 in regulating plant growth and development were initially characterized in *Arabidopsis thaliana*. For instance, AtBES1 improves seed yield and quality, mediates blue light-induced flowering responses, and modulates root foraging under low nitrogen [16,17,18]. The functional conservation and diversification of BES1/BZR1 homologs have been extensively validated across plant species. In tomato, BZR1.3 interacts with JMJ10, an H3R2me2a demethylase, to coordinate the epigenetic regulation of fruit size [19]. Rice OsBZR1 promotes amylose synthesis by interacting with OsSLRL2 [20]. MpBES/BZR3 regulates the development of haploid reproductive organs in Marchantia polymorpha [21]. Beyond developmental control, BES1/BZR1 TFs serve as crucial integrators of plant abiotic stress signaling. AtBES1 positively regulates plant thermotolerance and drought tolerance through physical interactions with stress-associated TFs, including heat shock factors (HSFs) and drought-responsive regulators RD26 and WRKY54 [13,22,23]. Additionally, AtBES1/BZR1 homolog 3 (*BEH3*) interacts with E3 ubiquitin ligase AtRZF1 to negatively regulate dehydration and abscisic acid (ABA) signaling pathways [24]. CsBES1.14, GmBES1.5, and TaBZR2 are reported to enhance cold, heat, and drought stress tolerance in tea, soybean, and wheat, respectively, by modulating transcription of downstream stress-responsive genes [25,26,27]. Collectively, these results reveal that BES1/BZR1 TFs exert versatile functions in coordinating plant growth, developmental progression, and abiotic stresses.

Maize is an essential multi-purpose crop, serving as a source of food, economic value, and feed. However, rapid socioeconomic development, ecological deterioration, and water scarcity have led to a progressive decline in maize yield. Consequently, dissecting the molecular mechanisms underlying stress tolerance in maize has become imperative [28]. In our previous study, we identified eleven ZmBES1/BZR1 TFs within the maize genome [29]. Among them, ZmBES1/BZR1-1, -3, -4, and -9 negatively regulate drought stress responses, respectively. Nevertheless, ZmBES1/BZR1-5 positively regulates drought tolerance and kernel size [30,31,32,33,34,35]. Moreover, ZmBES1/BZR1-4 negatively regulates seed development [32]. ZmBES1/BZR1-9 accelerates flowering in transgenic *Arabidopsis* and rice [33], suggesting the functional diversity of maize ZmBES1/BZR1 TFs. However, the function of ZmBES1/BZR1-7 remains largely unknown. In this study, we cloned and functionally characterized the *ZmBES1/BZR1-7* gene, analyzing its subcellular localization and expression pattern under drought stress. Furthermore, the potential roles of *ZmBES1/BZR1-7* in regulating drought tolerance were investigated via ectopic expression in *Arabidopsis* and rice. Collectively, this study advances our understanding of the molecular mechanisms by which ZmBES1/BZR1-7 governs drought stress response.

## 2. Results

### 2.1. ZmBES1/BZR1-7 Is a Typical BES1/BZR1 TF

**Figure 1:**
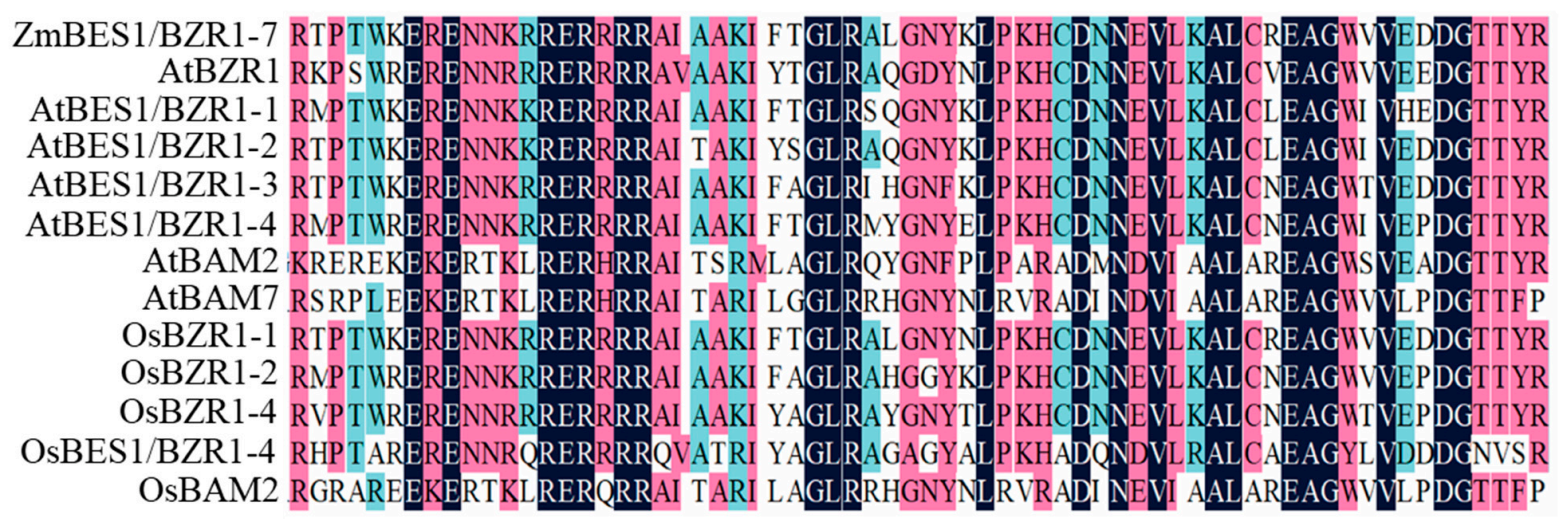
Sequence alignment of bHLH domain of ZmBES1/BZR1-7 and BES1/BZR1 proteins from *Arabidopsis* and *Oryza sativa*. The mazarine, pink, and light-blue backgrounds indicate perfect (100%), high (75%), and low (50%) conservation, respectively. The BES1/BZR1 amino acid sequences of *Oryza sativa* and *Arabidopsis thaliana* were obtained from the NCBI database. The name and accession number of each BES1/BZR1 sequence was OsBZR1-1 (NP_001409174.1), OsBZR1-2 (NP_001395971.1), OsBZR1-4 (XP_052141361.1), OsBES1/BZR1-4 (XP_025879038.2), OsBAM2 (XP_015611670.2), AtBES1/BZR1-1 (NP_190644.1), AtBES1/BZR1-2 (NP_195396.4), AtBES1/BZR1-3 (NP_193624.1), AtBES1/BZR1-4 (NP_565187.1), AtBZR1 (NP_565099.1), AtBAM2 (NP_199343.1), and AtBMY7 (NP_182112.2).

The CDS of ZmBES1/BZR1-7 is 975bp in length and encodes 324 amino acids with a molecular weight of 33.72 kDa, an isoelectric point (pI) of 8.47, a grand average of hydropathicity (GRAVY) index of −0.398, and an instability index of 65.61. Sequence alignment and phylogenetic analysis revealed that ZmBES1/BZR1-7 contained a highly conserved bHLH domain in the N-terminal region (Figure 1 and Figure S1) and showed high homology with rice OsBZR1-1 (Figure S2).

### 2.2. ZmBES1/BZR1-7 Is a Nuclear-Localization and Transcriptional Activator

**Figure 2:**
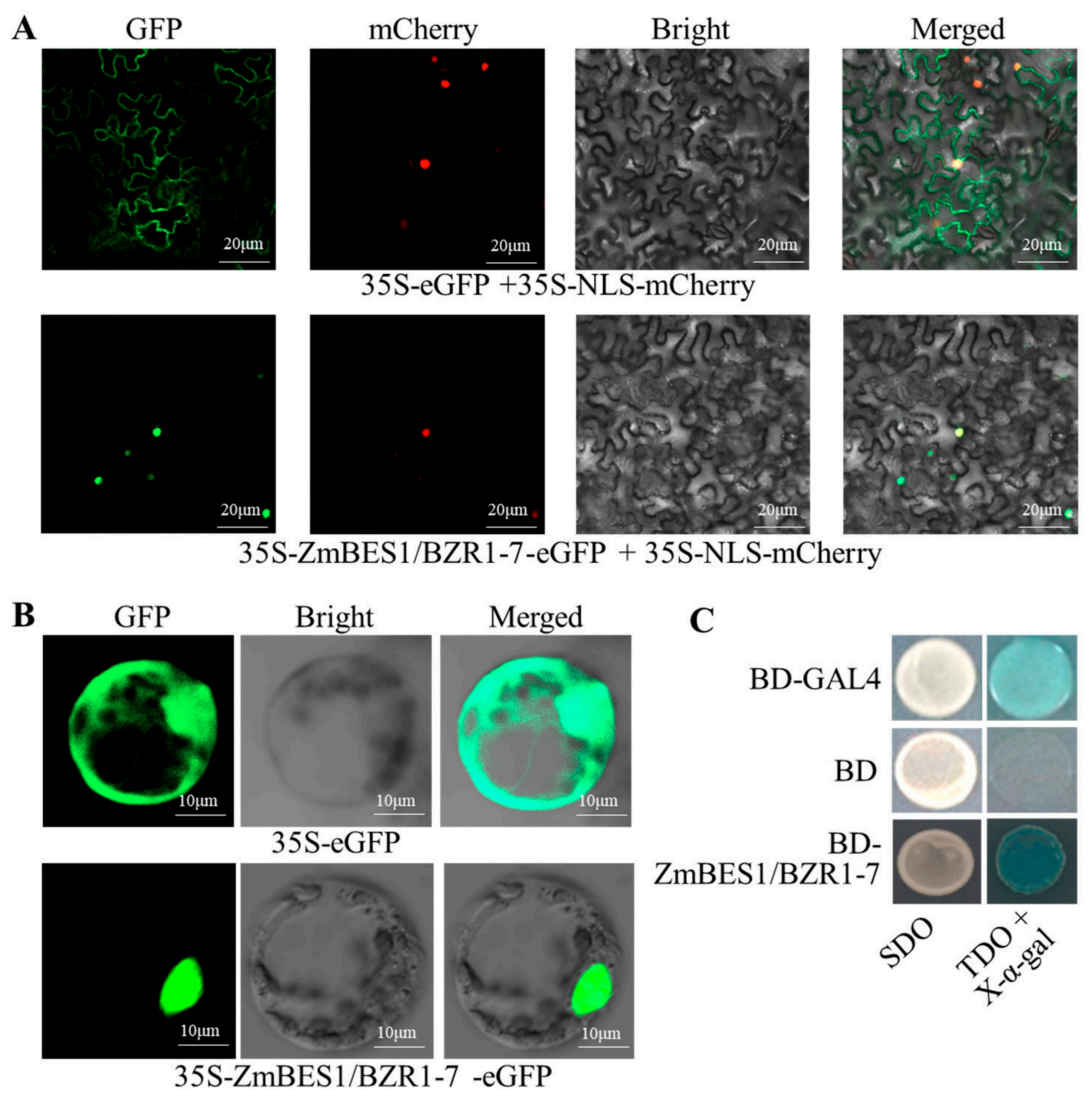
Subcellular localization and transcriptional activity analysis of ZmBES1/BZR1-7. (**A**) Localization of ZmBES1/BZR1-7 in *N. benthamiana* leaves. Scale bar = 20 μm. (**B**) Localization of ZmBES1/BZR1-7 in maize protoplasts. Scale bar = 10 μm. (**C**) Transcriptional activity assay of ZmBES1/BZR1-7. SDO, single dropout (Trp) plates. TDO, triple dropout (Trp/His/Ade) plates with X-α-Gal.

Subcellular localization analysis of ZmBES1/BZR1-7 was performed using a transient transformation system in *N. benthamiana* leaves. Leaves were co-transformed with 35S-eGFP plus 35S-NLS-mCherry (control) and 35S-ZmBES1/BZR1-7-eGFP plus 35S-NLS-mCherry. The results showed that the GFP fluorescence signal in leaves transformed with the 35S-eGFP empty vector was distributed throughout the entire cell. In contrast, the GFP fluorescence in the leaves transformed by the 35S-ZmBES1/BZR1-7-eGFP with the 35S-NLS-mCherry was observed exclusively in the nucleus (Figure 2A). The 35S-NLS-mCherry was used as a nuclear marker. Subsequently, to further verify the subcellular localization of the ZmBES1/BZR1-7 protein, the plasmids 35S-eGFP and 35S-ZmBES1/BZR1-7-eGFP were transformed into maize protoplasts, respectively. As shown in Figure 2B, the fluorescent signal of the ZmBES1/BZR1-7-eGFP fusion driven by the 35S promoter accumulated specifically in the nucleus. By comparison, the empty vector 35S-eGFP control showed uniform fluorescence covering the cytoplasm and nucleus. These results demonstrate that ZmBES1/BZR1-7 is a nuclear-localized protein.

To determine whether ZmBES1/BZR1-7 acts as a transcriptional activator, the yeast strain AH109 was transformed separately with pGBKT7-ZmBES1/BZR1-7, the pGBKT7-GAL4 (positive control), and the pGBKT7 (negative control). As shown in Figure 2C, all transformants grew normally on SDO plates, verifying successful transformation. On the QDO plates containing X-α-gal, only yeasts expressing pGBKT7-ZmBES1/BZR1-7 or pGBKT7-GAL4 were able to grow and develop blue colonies. Collectively, these findings indicate that ZmBES1/BZR1-7 exhibits transcriptional activity in yeast.

### 2.3. ZmBES1/BZR1-7 Transcription Is Responsive to Drought Stress

**Figure 3:**
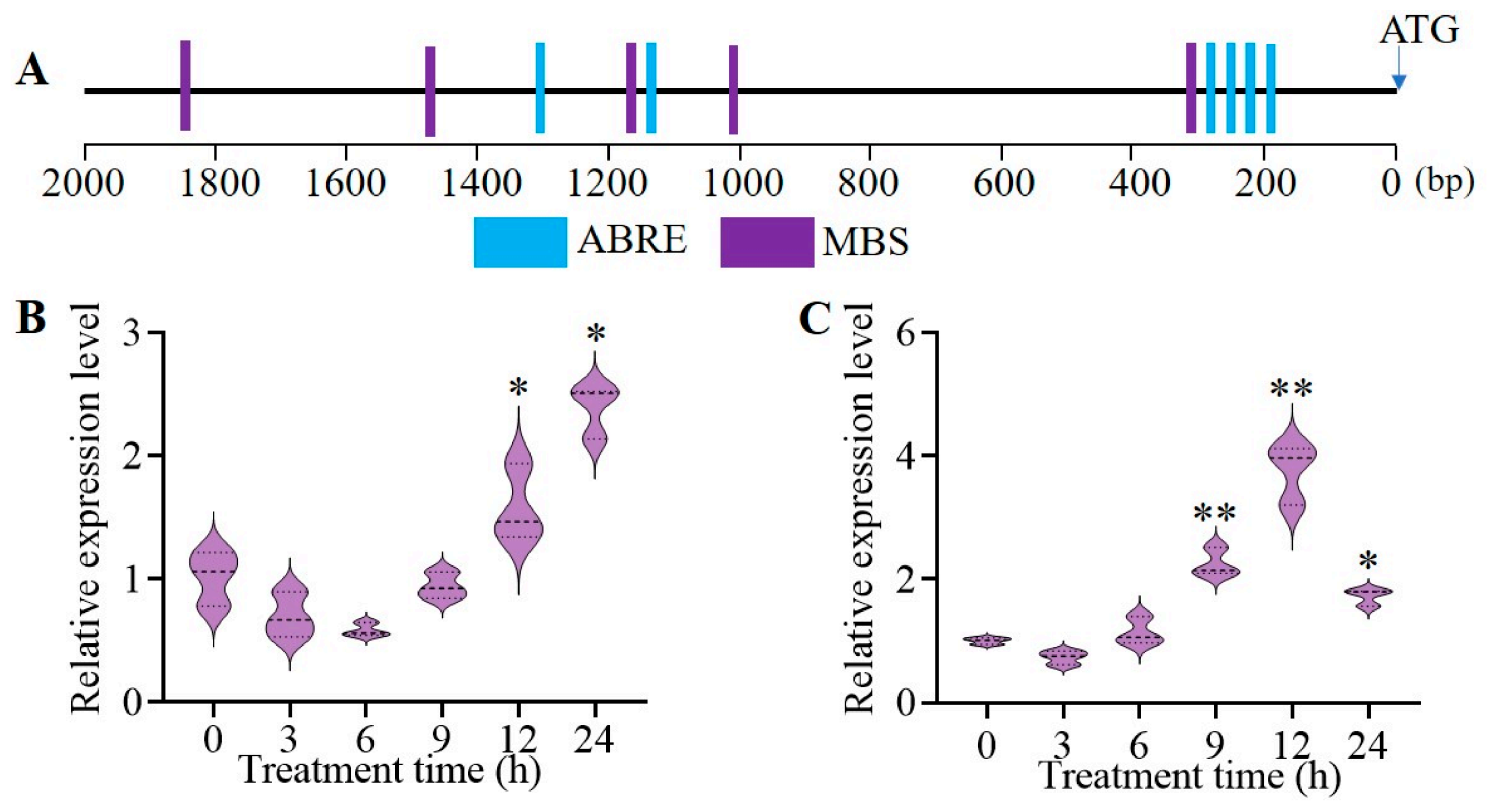
Response of *ZmBES1/BZR1-7* to drought stress. (**A**) cis-acting elements in the promoter region of *ZmBES1/BZR1-7*. MBS, MYB-binding site. ABRE, ABA-responsive element. (**B**,**C**) Relative expression level of *ZmBES1/BZR1-7* under PEG-mimicking drought stress in leaf (**B**) and root (**C**). * and ** represent significance at *p* < 0.05 and *p* < 0.01 levels compared to 0 h of treatment, respectively.

To identify potential regulatory elements governing *ZmBES1/BZR1-7* expression, the 2000 bp sequence upstream of its transcription start site was retrieved from MaizeGDB and used to analyze cis-acting elements using plantCARE. The results demonstrated that the promoter harbors several stress-responsive cis-elements, including five MBS motifs (MYB-binding site) associated with drought signaling and six ABREs (ABA-responsive elements) (Figure 3A), suggesting that *ZmBES1/BZR1-7* may participate in the drought stress response. To further examine the transcription level of *ZmBES1/BZR1-7* under drought stress, qRT-PCR was conducted to assess *ZmBES1/BZR1-7* transcript levels under PEG-induced drought stress. Notably, *ZmBES1/BZR1-7* was significantly induced in both leaves and roots (Figure 3B,C), suggesting that *ZmBES1/BZR1-7* is responsive to drought.

### 2.4. Overexpression of ZmBES1/BZR1-7 Improves Drought Tolerance in Transgenic Arabidopsis

**Figure 4:**
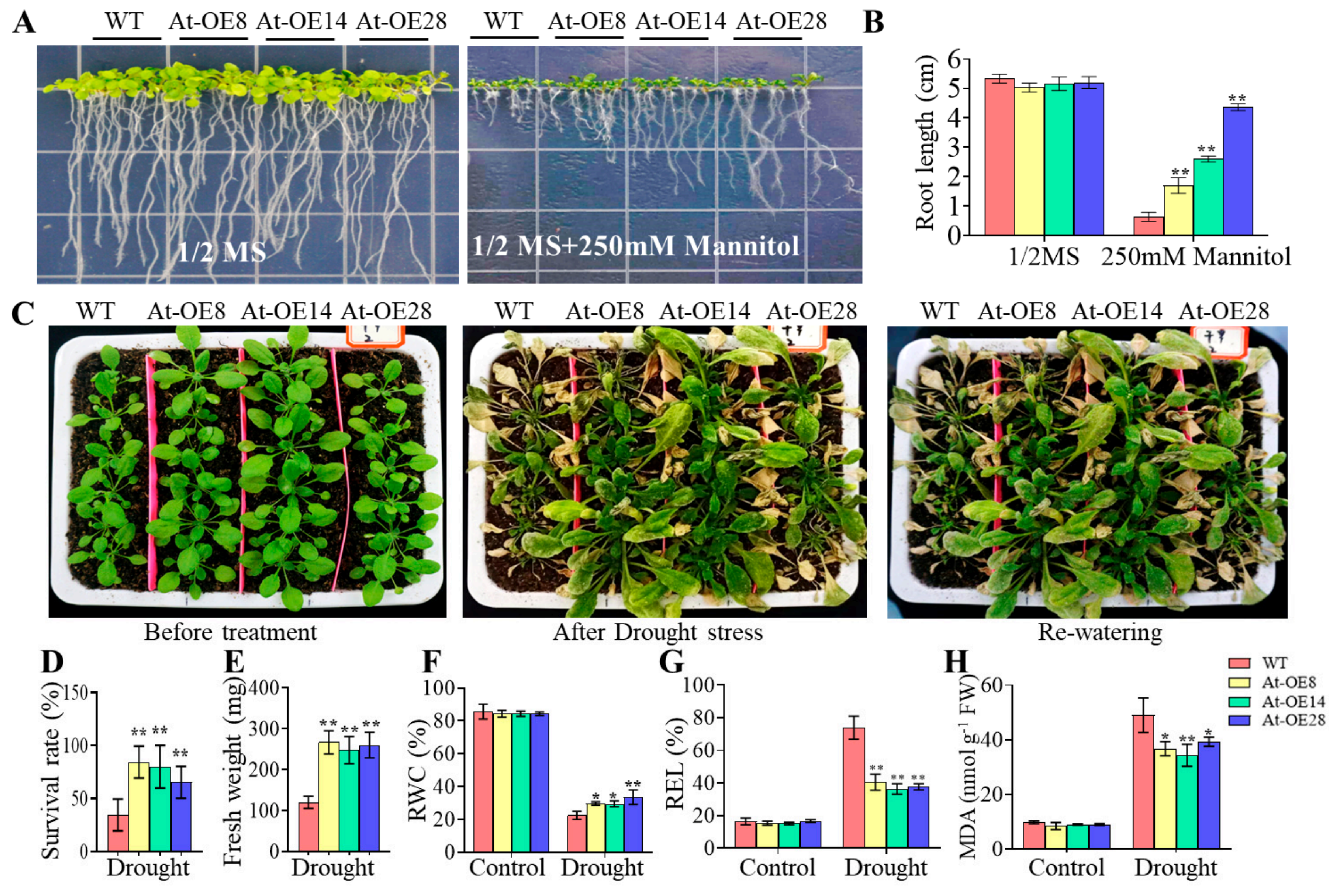
Ectopic expression of *ZmBES1/BZR1-7* improves drought tolerance of transgenic *Arabidopsis*. (**A**,**B**) Root phenotype (**A**) and statistical data of root length (**B**) of transgenic *Arabidopsis* lines and WT on 1/2 MS without (control) and with 250 mM mannitol plates. (**C**) The phenotypes of all seedlings were recorded before and after 14 days of drought treatment. (**D**–**H**) Survival rate (**D**), fresh weight (**E**), relative water content (RWC) (**F**), relative electrolyte leakage (REL) (**G**), and malondialdehyde (MDA) content (**H**) of all seedlings after drought stress. WT, wild type. At-OE8, At-OE14 and At-OE28, homozygous transgenic *Arabidopsis* lines expressing *ZmBES1/BZR1-7*. * and ** mean *p* < 0.05 and <0.01, respectively.

To confirm the biological function of *ZmBES1/BZR1-7* in mediating drought tolerance, three homozygous lines of *Arabidopsis* (At-OE8, At-OE14, At-OE28) were generated, screened, and identified at the DNA, transcription, and protein levels, through PCR amplification, qRT-PCR, and ZmBES1/BZR1-7-GFP imaging, respectively (Figure S3). Hence, they were used for phenotyping under drought stress treatment. As shown in Figure 4, on 1/2 MS plates (control), the At-OE8, At-OE14, and At-OE28 lines showed no difference compared to WT. However, on 1/2 MS medium supplemented with 250 mM mannitol, three transgenic lines exhibited superior growth, longer roots, and higher fresh weight compared to WT. Meanwhile, three-week-old seedlings were cultivated in soil under greenhouse conditions for phenotyping. The results showed that there were no significant differences in growth between transgenic lines and WT under well-watered conditions. However, under drought stress, all plants exhibited impaired growth, while the At-OE8, At-OE14, and At-OE28 lines exhibited superior growth compared to WT. After 3 days of re-watering, the survival rates of all transgenic lines were significantly higher than those of WT. Furthermore, compared with WT, transgenic lines showed significantly higher RWC but lower REL and MDA contents. This suggests that *ZmBES1/BZR1-7* plays a positive role in enhancing drought tolerance in transgenic *Arabidopsis*.

### 2.5. Overexpression of ZmBES1/BZR1-7 Enhances Drought Tolerance in Transgenic Rice

**Figure 5:**
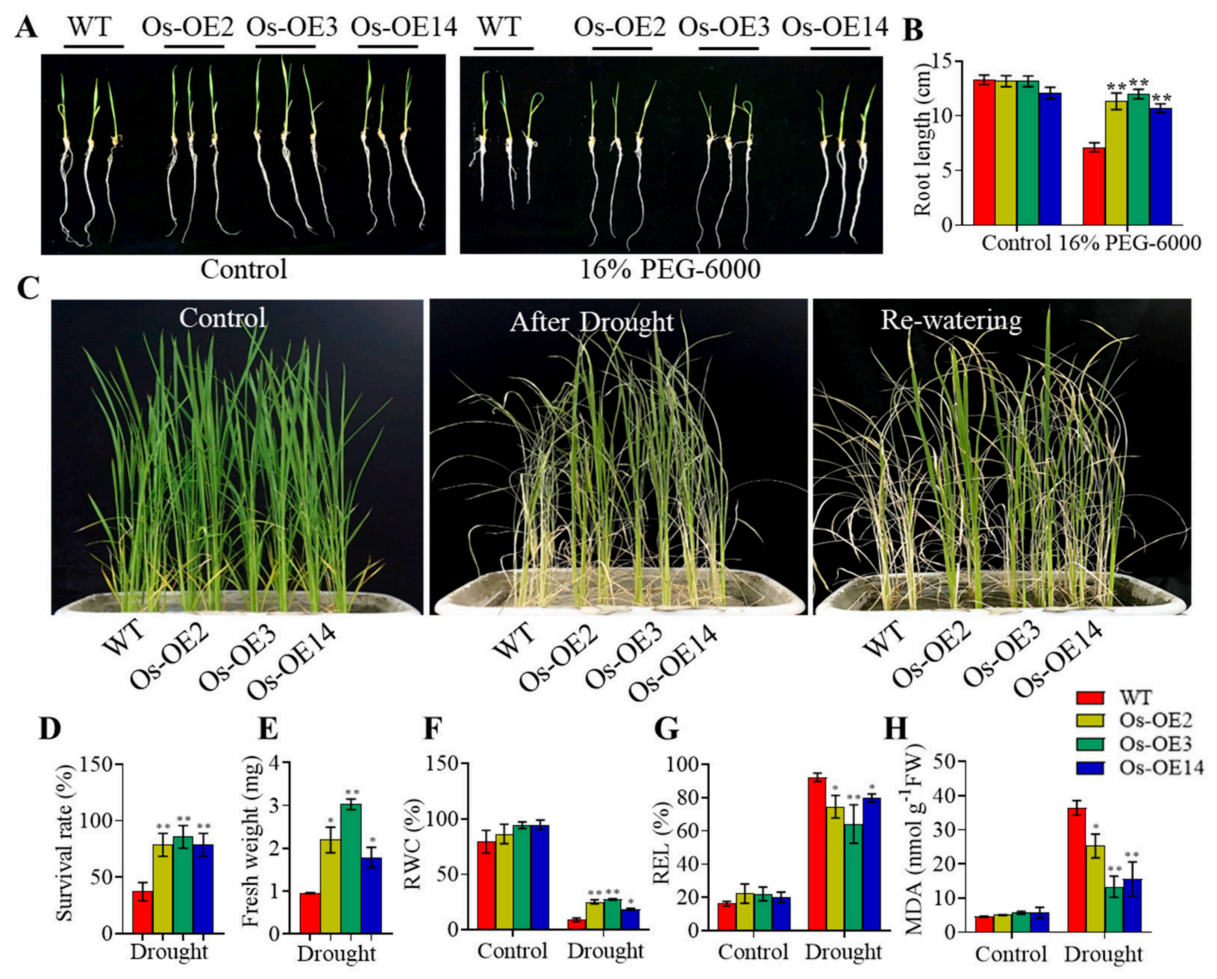
Overexpression of *ZmBES1/BZR1-7* improves drought tolerance of transgenic rice seedlings. (**A**,**B**) Root phenotype (**A**) and root length (**B**) of transgenic rice and WT before and after 16% PEG-6000 treatment. (**C**) Phenotype of all seedlings before and after 10 days of drought treatment. (**D**–**H**) Survival rate (**D**), fresh weight (**E**), relative water content (RWC) (**F**), relative electrolyte leakage (REL) (**G**), and malondialdehyde (MDA) content (**H**) of all lines after drought treatment. WT, wild type. Os-OE2, Os-OE3 and Os-OE14, transgenic rice lines overexpressing *ZmBES1/BZR1-7*. * and ** represent *p* < 0.05 and 0.01, respectively.

To further confirm that *ZmBES1/BZR1-7* positively regulates drought tolerance, three homozygous transgenic rice lines expressing *ZmBES1/BZR1-7* were generated, as confirmed by PCR, qRT-PCR, and GFP imaging analysis at the DNA, transcription, and protein levels, defined as Os-OE2, Os-OE3, and Os-OE14, respectively, and used for phenotyping (Figure S4). The results showed that seedlings of Os-OE2, Os-OE3, and Os-OE14 lines displayed longer roots than WT after 7 days of treatment using 16% PEG-6000 conditions (Figure 5A,B). Meanwhile, five-leaf-old seedlings of all plants were subjected to water withholding for stress. As shown in Figure 5C, all plants exhibited a uniform growth phenotype prior to treatment. However, after drought stress, Os-OE2, Os-OE3, and Os-OE14 lines exhibited superior growth and light wilting symptoms, although all plants exhibited impaired growth. Furthermore, the survival rates, fresh weight, and RWC of all transgenic lines were significantly higher than the WT but REL and MDA content of transgenic lines were significantly lower compared to WT (Figure 5D–H).

**Figure 6:**
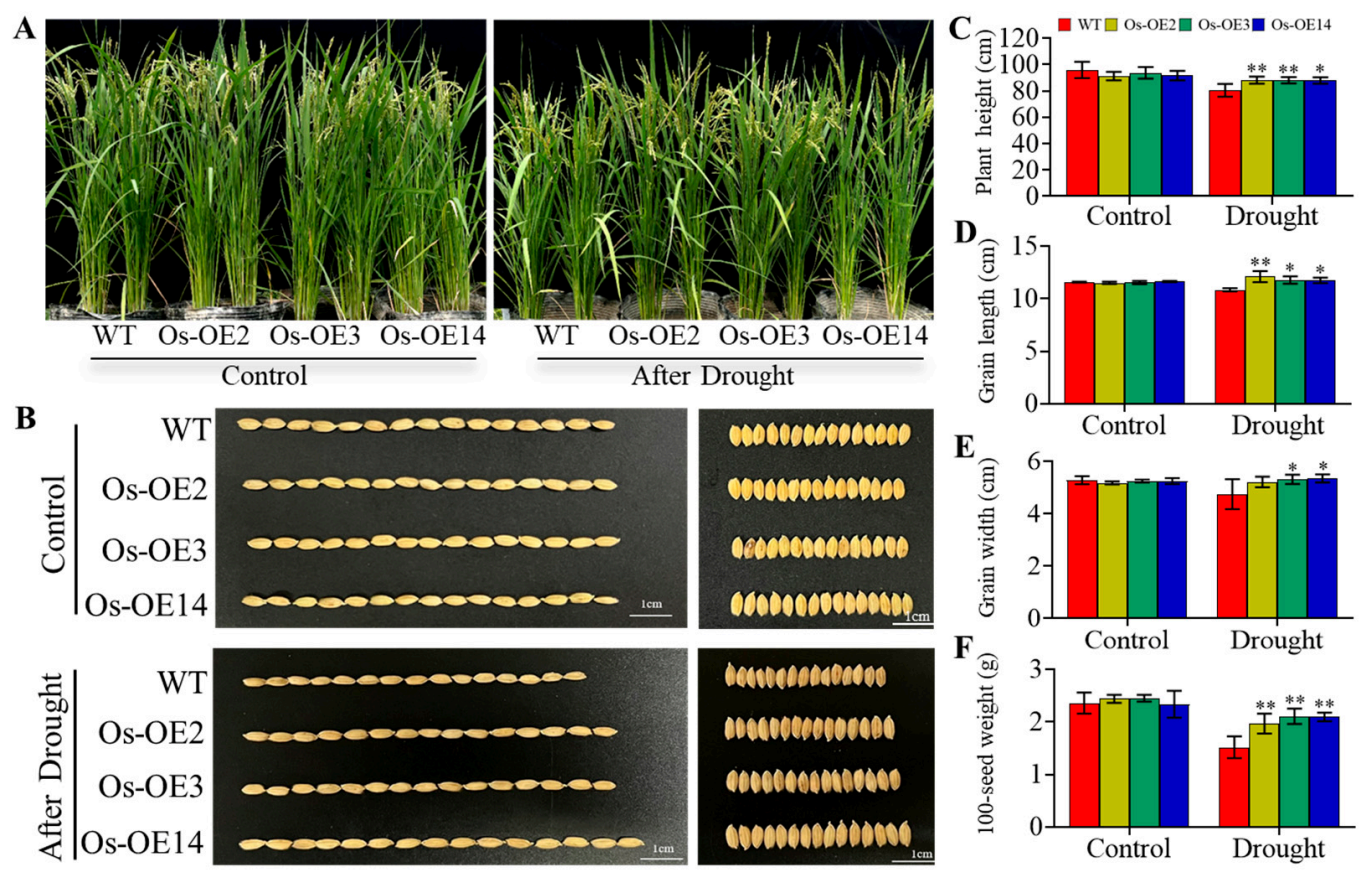
*ZmBES1/BZR1-7* enhances seed yield in transgenic rice. (**A**,**B**) Plant (**A**) and seed (**B**) phenotype of all seedlings under well water (control) and drought conditions. (**C**–**F**) Plant height (**C**), 15-seed length (**D**) and width (**E**), and 100-seed weight (**F**) of transgenic rice lines and WT. WT, wild type. Os-OE2, Os-OE3 and Os-OE14, transgenic rice lines overexpressing *ZmBES1/BZR1-7*. * and ** mean *p* < 0.05 and <0.01), respectively.

Moreover, all transgenic lines and WT were cultivated in soil and subjected to drought stress by withholding water during the heading stage for yield evaluation. As shown in Figure 6, there was no significant difference between *ZmBES1/BZR1-7*-transgenic lines and WT under natural conditions. However, under drought conditions, Os-OE2, Os-OE3, and Os-OE14 lines exhibited superior growth, higher plant height, longer grain length, wider grain width, and higher hundred-grain weight compared with WT.

### 2.6. ZmBES1/BZR1-7 Regulates Transcription of Stress-Responsive Genes

**Figure 7:**
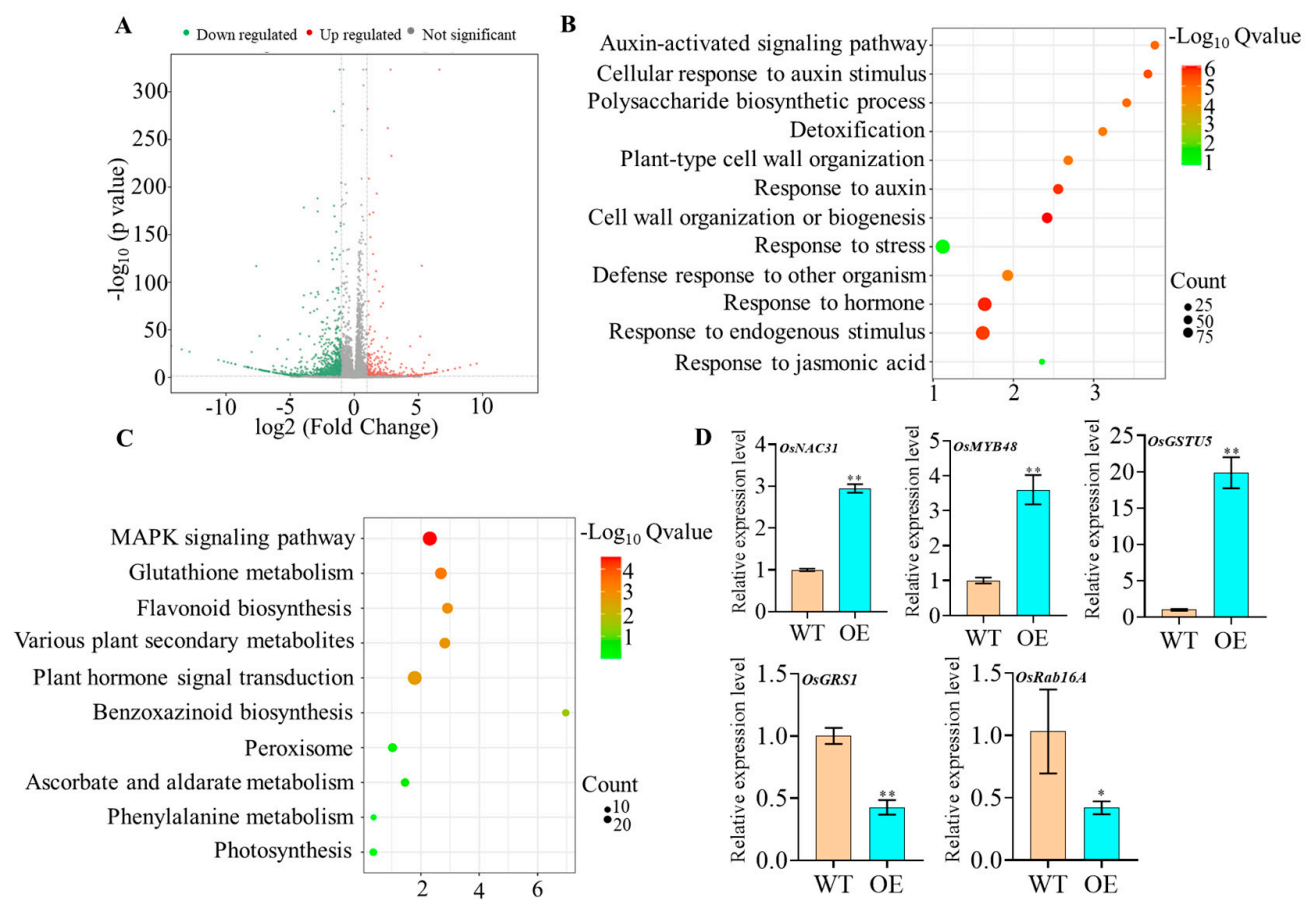
ZmBES1/BZR1-7 regulates transcription of stress-responsive genes. (**A**) Volcano plot of DEGs identified in transgenic rice. (**B**) GO enrichment analysis of DEGs. (**C**) KEGG enrichment analysis of DEGs. (**D**) qRT-PCR validation of DEGs. WT, wild type. OE, the mixtures of all transgenic lines. * and ** indicate *p* < 0.05 and <0.01), respectively.

To dissect the transcriptional regulatory network downstream of ZmBES1/BZR1-7, we performed RNA-sequencing (RNA-seq) analysis in rice lines overexpressing *ZmBES1/BZR1-7* and WT. In total, 1725 differentially expressed genes (DEGs) were identified in transgenic lines compared to WT, of which 396 DEGs were upregulated and 1329 DEGs were downregulated (Figure 7A). Gene ontology (GO) enrichment analysis demonstrated that 200 DEGs were significantly enriched in stress-response regulatory pathways (Figure 7B). Kyoto Encyclopedia of Genes and Genomes (KEGG) analysis corroborated that the DEGs were significantly enriched in the MAPK signaling pathway, glutathione metabolism, flavonoid biosynthesis, various plant secondary metabolites and plant hormone signal transduction pathway (Figure 7C). Notably, 5 DEGs, including *OsNAC31* (LOC_Os08g01330), *OsGSTU5* (LOC_Os09g20220), *OsMYB48* (LOC_Os01g74410), *OsGSR1* (LOC_Os06g15620), and *OsRab16A* (LOC_Os11g26790), have been reported to be involved in regulating abiotic stress, including drought, salinity, wounding, and ABA responses. The results of qRT-PCR confirmed that the expression of *OsNAC31*, *OsGSTU5*, and *OsMYB48* was upregulated in transgenic lines, whereas transcription of *OsGSR1* and *OsRab16A* was downregulated compared to WT (Figure 7D), which was consistent with the RNA-seq results. Collectively, these findings suggest that the heterologous expression of *ZmBES1/BZR1-7* in transgenic rice enhances drought stress tolerance by modulating the expression of stress-responsive genes.

## 3. Discussion

Due to high starch and nutrient content in the kernel, maize plays a significant role in food security and economic stability, particularly in developing countries. However, drought diminishes water availability, impairing photosynthesis and nutrient absorption, causing wilting and poor root development, and decreasing maize yields [36]. Hence, identifying stress-associated genes and elucidating their regulatory networks is essential for improving stress tolerance in crops [37]. Plant BES1/BZR1 TFs regulate growth, development, and stress responses [38,39]. Here, we found that ZmBES1/BZR1-7 possesses a highly conserved bHLH domain and is nuclear-localized and transcriptionally active (Figure 1 and Figure 2), confirming it as a canonical BES1/BZR1 TF.

Transcription of nuclear genes is regulated by diffusible trans-factors that recognize cis-regulatory elements in the gene promoter sequence and reveal potential functions [40]. Here, we found numerous stress-responsive elements, including five MBS and six ABRE, in the *ZmBES1/BZR1-7* promoter (Figure 3A). Previous studies have definitively verified that MBS (MYB-binding site) and ABRE (ABA-responsive element) serve as core *cis*-regulatory elements in plant stress transcriptional networks, which specifically function as the binding locus of MYB TFs and the essential responsive motif for ABA signaling, respectively; both elements are pivotal functional components governing plant drought stress adaptation and tolerance regulation [7,41,42]. Meanwhile, qRT-PCR results showed that drought stress induced the expression of *ZmBES1/BZR1-7* (Figure 3B,C), implying that *ZmBES1/BZR1-7* may play a role in drought stress response. Hence, *ZmBES1/BZR1-7* was ectopically expressed in *Arabidopsis* and rice, resulting in enhanced drought tolerance of transgenic lines with longer roots, higher fresh weight and RWC, lower REL and MDA content, as well as higher yield of transgenic rice (Figure 4, Figure 5 and Figure 6). It has been previously established that BES1/BZR1 TFs regulate root foraging and length, fiber length, and plant growth. For example, BES1 modulates brassinosteroid-regulated root forging response under low nitrogen in *Arabidopsis* [16]. Heterologous expression of the *ZmBES1/BZR1-1/-3/-9* gene in *Arabidopsis* could increase the drought sensitivity of transgenic lines, resulting in shorter roots, lower fresh weight, ROS levels, and RWC, as well as higher stomatal aperture and REL than WT [30,31]. The *det2-9* mutant, which is defective in brassinosteroid (BR) biosynthesis, exhibits a short-root phenotype resulting from reduced cell numbers in the root meristem and diminished cell size in the root maturation zone [43]. AtBZR1 binds to the promoter of the root stem cell master gene *AtPLT1* and represses its transcriptional activity, thereby modulating the primary root length in transgenic *Arabidopsis* [44]. Evidence from studies on birch, wheat, soybean, and tea has demonstrated that members of the BES1/BZR1 TFs are involved in signaling pathways triggered by multiple stresses, including stripe rust, salinity, cold, and heat, by modulating downstream gene transcription. GmBES1.5 directly binds to the E-box and activates abiotic stress-response genes to improve heat stress tolerance in soybean [26]. CsBES1-14 positively regulates cold resistance in tea plants by regulating the expression of *CsCOR413* in the tea plant [27]. TaBZR2 activates chitinase *Cht20.2* transcription to confer resistance to wheat stripe rust [45]. BpBZR1-6 positively regulates salt stress response by regulating the expression of *P5CSs* in birch [46]. In this study, 200 DEGs identified in the transgenic lines compared to the WT were significantly enriched in stress-response regulatory pathways (Figure 7B). Notably, five DEGs, including the upregulated genes *OsNAC31*, *OsGSTU5*, and *OsMYB48*, and the downregulated genes *OsGSR1* and *OsRab16A*, were identified (Figure 7D). These genes have been previously confirmed to regulate drought, salt, and ABA responses. OsNF-YC enhances drought and salt tolerance in transgenic rice by regulating the expression of *OsRab16A* [47]. OsWRKY12 negatively regulates drought stress tolerance and secondary cell wall biosynthesis by targeting *OsNAC31* in rice [48]. ABI3 enhances plant salt tolerance through the regulation of the brassinosteroid biosynthesis gene *OsGSR1* [49]. Overexpression of *OsMYB48* enhances drought and salinity tolerance in rice [50]. ZmBES1/BZR1-1 and -3 were also reported to negatively regulate drought tolerance by regulating oxidative stress-related gene expression [30,31], suggesting functional diversity among the ZmBES1/BZR1 family. Hence, the function of *ZmBES1/BZR1-7* will be verified using endogenous expression and knockout in maize in future studies.

In conclusion, the expression of *ZmBES1/BZR1-7* is regulated by drought. Ectopic expression of *ZmBES1/BZR1-7* improves drought tolerance of transgenic *Arabidopsis* and rice by regulating stress-responsive gene transcription. Our study provides insights for further investigating the roles of ZmBES1/BZR1s in the maize stress response.

## 4. Materials and Methods

### 4.1. Plant Materials and Growth Conditions

According to the methods reported in our previous studies [31,34,51], seeds of *Arabidopsis thaliana* (Col-0), *Nicotiana benthamiana*, *Oryza sativa* (Nipponbare), and *Zea mays* (B73 inbred line) were germinated and grown in a greenhouse under 14 h light (26 °C)/10 h dark (22 °C) conditions. In this study, *ZmBES1/BZR1-7* overexpression lines were generated in *Arabidopsis* and rice, and while maize B73 seedlings were used to analyze the expression of *ZmBES1/BZR1-7*. For drought stress treatment, three-leaf-old B73 seedlings were exposed to a 16% PEG-6000 solution. Shoots and roots samples were collected at 0, 3, 6, 12, and 24 h after treatment for RNA extraction.

### 4.2. Sequence Analysis

The genomic DNA (gDNA) sequence, coding sequence (CDS), and amino acid sequence of ZmBES1/BZR1-7 were downloaded from the MaizeGDB database (http://maizegdb.org/, accessed on 5 May 2023). The physicochemical properties of the ZmBES1/BZR1-7 protein were analyzed by the ProtParam (http://web.expasy.org/protparam/, accessed on 7 May 2023). The amino acid sequences of BES1/BZR1 from *Oryza sativa* and *Arabidopsis thaliana* were obtained from NCBI (https://www.ncbi.nlm.nih.gov/genbank/, accessed on 5 May 2026) and used for multiple alignment with ZmBES1/BZR1-7 using DNAMAN v6 software. Phylogenetic analysis was performed using MEGA7.0, in which the tree was generated using the Neighbor Joining algorithm with 1000 bootstrap replicates.

### 4.3. Quantitative Real-Time PCR (qRT-PCR)

The total RNA was extracted from the above samples using Trizol reagent (Takara, Dalian, China) following the protocol reported by Feng et al. [31] and used for cDNA synthesis by the ABScript III RT Master Mix for qPCR with gDNA Remover kit (ABclonal, Wuhan, China). qRT-PCR was conducted on a Bio-Rad CFX96™ Real-Time PCR (Bio-Rad, Hercules, CA, USA) system using 2× Universal SYBR Green Fast qPCR Mix (ABclonal, Wuhan, China), as described by Feng et al. [31] and Sun et al. [34]. Additionally, the fragment of the *ZmGAPDH* gene was amplified to serve as an internal reference control. The primers are listed in Table S1.

### 4.4. Subcellular Localization

To construct the *35S-ZmBES1/BZR1-7-eGFP* vector for subcellular localization detection, Primer 5.0 was used to design specific primers amplifying the stop codon-truncated CDS of *ZmBES1/BZR1-7* (Table S1). The *pCAMBIA-35S-eGFP* plasmid was linearized by *BamH* I and *Hind* III (TaKaRa, Dalian, China), and the PCR product was cloned into the linearized *pCAMBIA-35S-eGFP* plasmid using the ClonExpress^®^ II One Step Cloning Kit (Vazyme, Nanjing, China) to generate the *35S-ZmBES1/BZR1-7-eGFP* construct. As previously established [51], the *35S-ZmBES1/BZR1-7-eGFP* plasmid was transformed into Agrobacterium GV3101 for transient expression in *N. benthamiana* leaves. Meanwhile, the *35S-ZmBES1/BZR1-7-eGFP* plasmid was transformed into maize protoplasts. After injection and transformation, the tobacco and maize protoplasts were cultured for 36–48 h and 12–16 h at 28 °C, respectively, and then used for fluorescence imaging using a confocal microscope LSM800 (Carl Zeiss, Oberkochen**,** Germany). *35S-NLS-mCherry* and *35S-eGFP* were used as controls.

### 4.5. Transcriptional Activity Analysis

To assess the transcriptional activity of ZmBES1/BZR1-7, specific primers were designed to amplify its CDS (Table S1). The amplified PCR products were inserted into the *pGBKT7* vector with the DNA-binding domain (BD) to construct the *BD-ZmBES1/BZR1-7* recombinant vector. Following the protocol described by Ren et al. [52], the constructs *BD-ZmBES1/BZR1-7*, *BD-GAL4* (positive control), and *BD* (negative control) were transformed into the yeast strain AH109. The transformed yeast cells were streaked onto single dropout (Trp) (SDO) and triple dropout (Trp/His/Ade) (TDO) medium plates supplemented with X-α-gal, followed by incubation at 28 °C for 3 days.

### 4.6. Plant Transformation

For stable genetic transformation, *Agrobacterium tumefaciens* strain GV3101 harboring the recombinant *35S-ZmBES1/BZR1-7-eGFP* plasmid was employed. *Arabidopsis thaliana* (Col-0) and rice (Nipponbare) were transformed via floral-dipping and *Agrobacterium*-mediated embryo transformation, respectively, as described previously [53,54]. Transgenic lines were screened with 50 mg/L kanamycin over successive generations to obtain homozygous lines. The positive transgenic lines were further validated at the DNA, RNA, and protein levels using PCR, qRT-PCR and GFP fluorescence imaging, respectively. The primers for PCR and qRT-PCR are listed in Table S1. Root tips from homozygous lines were examined for eGFP signals under a confocal microscope (Carl Zeiss, Oberkochen, Germany).

### 4.7. Drought Phenotyping Assay

Following the methods described by Feng et al. [30], seeds of transgenic *Arabidopsis* and wild type (WT) were surface-sterilized and germinated on 1/2 MS solid medium. For drought stress treatment, 1/2 MS plates supplemented with 250 mM mannitol were prepared as the stress group, while standard 1/2 MS medium served as the control. After two weeks of cultivation, seedling phenotypes were captured by photography, and the root length as well as the fresh weight of each genotype were quantified. For soil-based drought assays, three-week-old *Arabidopsis* seedlings were grown in soil and subjected to drought treatment by withholding water. When leaves of WT or transgenic lines showed complete wilting, the plants were kept under dehydration for three days and rewatered for one week. The survival rate, fresh weight, RWC, REL, and MDA content were measured following the methods described by Yu et al. [51].

For phenotyping of transgenic rice, after germination, transgenic lines and WT were divided into three batches. One batch was subjected to 16% PEG-6000 treatment, while the other two batches were planted in soil for drought treatment by withholding water during the vegetative and reproductive growth stages, respectively. After seven days of 16% PEG-6000 treatment, root length was measured for each line. Meanwhile, five-leaf-old seedlings grown in soil were subjected to water withholding for 10 days and then rewatered for recovery over three days. The survival rate, fresh weight, RWC, REL, and MDA content of each line were measured. To evaluate seed yield under drought stress, seedlings of transgenic rice and WT were planted in a greenhouse and subjected to water withholding during the heading and grain-filling stages, following the methods described by Lu et al. [55]. Finally, the plant height, grain length, grain width, and 100-seed weight were measured for each line.

### 4.8. RNA-Sequencing (RNA-Seq) and qRT-PCR

As previously described in our studies [51], two-week-old seedlings of transgenic rice lines and WT were subjected to 16% PEG-6000 treatment. Seedling samples were subsequently collected for total RNA extraction and transcriptome sequencing, which was performed by Sanshu Biotechnology Co., Ltd. (Nantong China). RNA quality and integrity were evaluated using a Bioanalyzer 2100 (Agilent, Santa Clara, CA, USA). The sequencing library was constructed with the VAHTSTM mRNA-seq V2 Library Prep Kit and sequenced on an Illumina NovaSeq 6000 platform (IIIumina, San Diego, CA, USA). The sequencing data were analyzed by quality controlling, adapter filtering, aligning to the rice genome, transcript assemble, and expression quantification using FastQC (v0.11.2), Trimmomatic (v0.36), HISAT2 (v2.2.1), and StringTie (v3.0.0), respectively, to identify DEGs between transgenic lines and WT with a threshold of *p*-value < 0.05 and |log2 Fold Change| ≥ 1. GO and KEGG enrichments were analyzed using R package clusterProfiler (v4.17.0). qRT-PCR analysis was performed to verify the expression patterns of candidate DEGs, with all validation primers listed in Table S1.

### 4.9. Statistical Analysis

Each experiment was carried out in three independent replicates. Results are expressed as the mean ± standard deviation (SD). Statistical significance was assessed by one-way ANOVA, where differences at *p* < 0.05 (*) and *p* < 0.01 (**) were deemed statistically significant.

## 5. Conclusions

In summary, *ZmBES1/BZR1-7* acts as a positive regulator of drought tolerance. Overexpression of *ZmBES1/BZR1-7* improved drought resistance in transgenic *Arabidopsis* and rice lines, which exhibited longer roots, greater fresh weight and RWC, reduced REL and MDA accumulation, alongside higher grain yield of transgenic rice under drought conditions. We further demonstrated that ZmBES1/BZR1-7 enhances drought tolerance in transgenic plants by modulating the transcript levels of stress-responsive genes, including *OsNAC31*, *OsGSTU5*, *OsMYB48*, *OsGSR1*, and *OsRab16A*. These findings provide a foundation for elucidating the mechanistic roles of *ZmBES1/BZR1-7* in regulating maize adaptation to drought stress response. However, the biological function of ZmBES1/BZR1-7 still need to be further validated using endogenous functional analyses and maize knockout mutants in future investigations.

## Data Availability

The original contributions presented in this study are included in the article/Supplementary Materials. Further inquiries can be directed to the corresponding author.

## Supplementary Materials

The following supporting information can be downloaded at: https://www.mdpi.com/article/10.3390/plants15152359/s1, Figure S1. Sequence alignment of ZmBES1/BZR1-7 and BES1/BZR1 proteins from *Arabidopsis* and Oryza sativa. Figure S2. Phylogenic tree of ZmBES1/BZR1-7 and BES1/BZR1 proteins in *Arabidopsis* and rice. Figure S3. Identification of Arabidopsis transgenic lines overexpression of the *ZmBES1/BZR1-7* gene. Figure S4. Identification of rice transgenic lines overexpression of the *ZmBES1/BZR1-7* gene. Table S1. Primers used in this study.
