# Supplementary material for: Maize ZmBES1/BZR1-7 Transcription Factor Positively Regulates Drought Tolerance in Transgenic Arabidopsis and Rice
Source: Plants (Basel). 2026 Jul 31;15(15):2359. doi: 10.3390/plants15152359 (PMC13468094; doi:10.3390/plants15152359)
Supplement: Supplementary file 1 [file plants-15-02359-s001.zip › supplementary data.pdf]

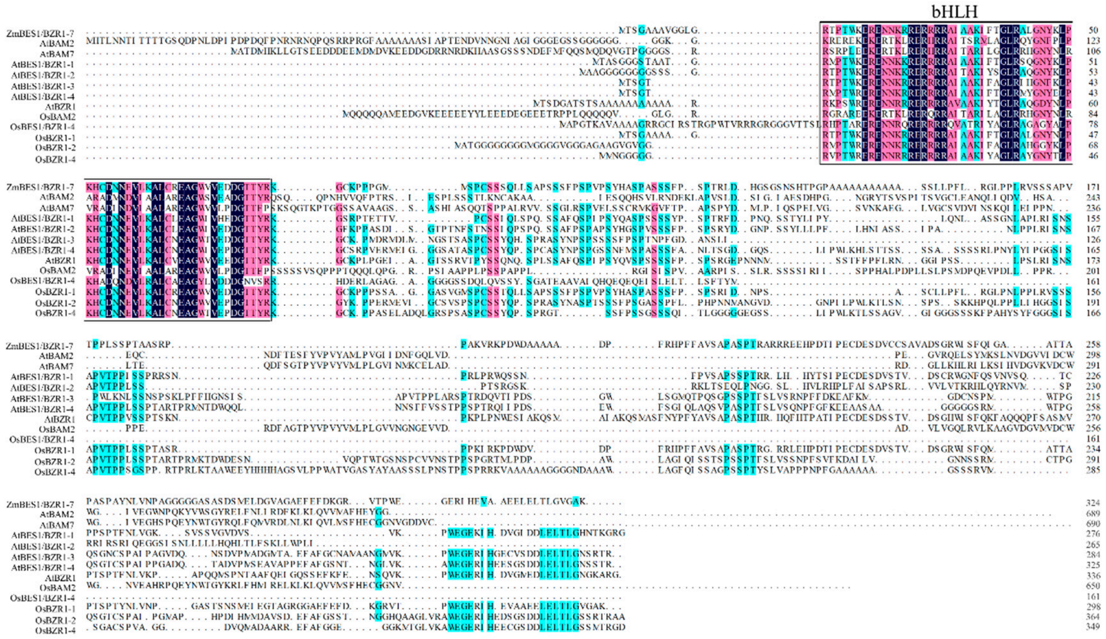

Figure S1. Sequence alignment of ZmBES1/BZR1-7 and BES1/BZR1 proteins from *Arabidopsis* and Oryza sativa. Box, bHLH domain. The BES1/BZR1 amino acid sequences of Oryza sativa and *Arabidopsis thaliana* were obtained from NCBI database. The name and accession number of each BES1/BZR1 sequence was OsBZR1-1 (NP_001409174.1), OsBZR1-2 (NP_001395971.1), OsBZR1-4 (XP_052141361.1), OsBES1/BZR1-4 (XP_025879038.2), OsBAM2 (XP_015611670.2), AtBES1/BZR1-1 (NP_190644.1), AtBES1/BZR1-2 (NP_195396.4), AtBES1/BZR1-3 (NP_193624.1), AtBES1/BZR1-4 (NP_565187.1), AtBZR1 (NP_565099.1), AtBAM2 (NP_199343.1), and AtBMY7 (NP_182112.2).

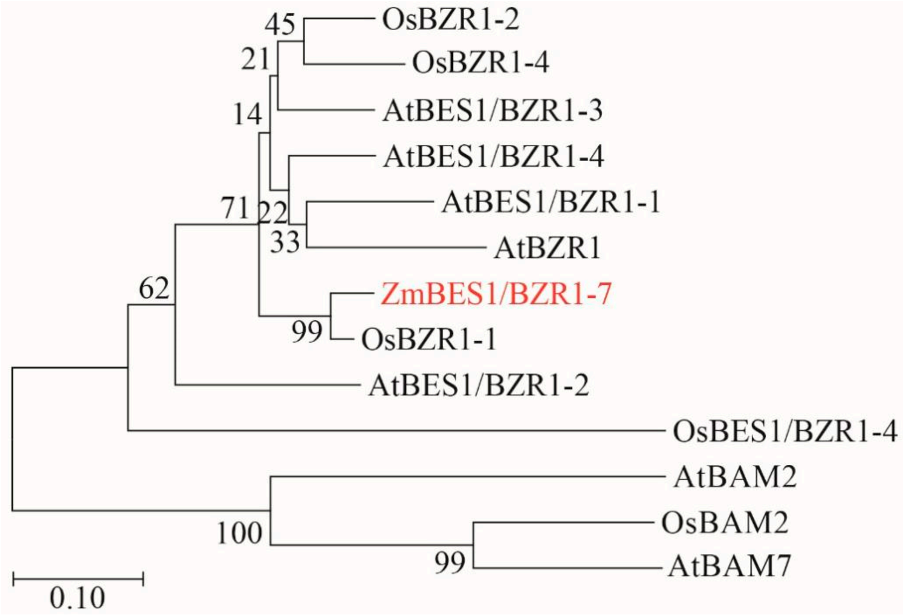

Figure S2. Phylogenic tree of ZmBES1/BZR1-7 and BES1/BZR1 proteins in *Arabidopsis* and rice.

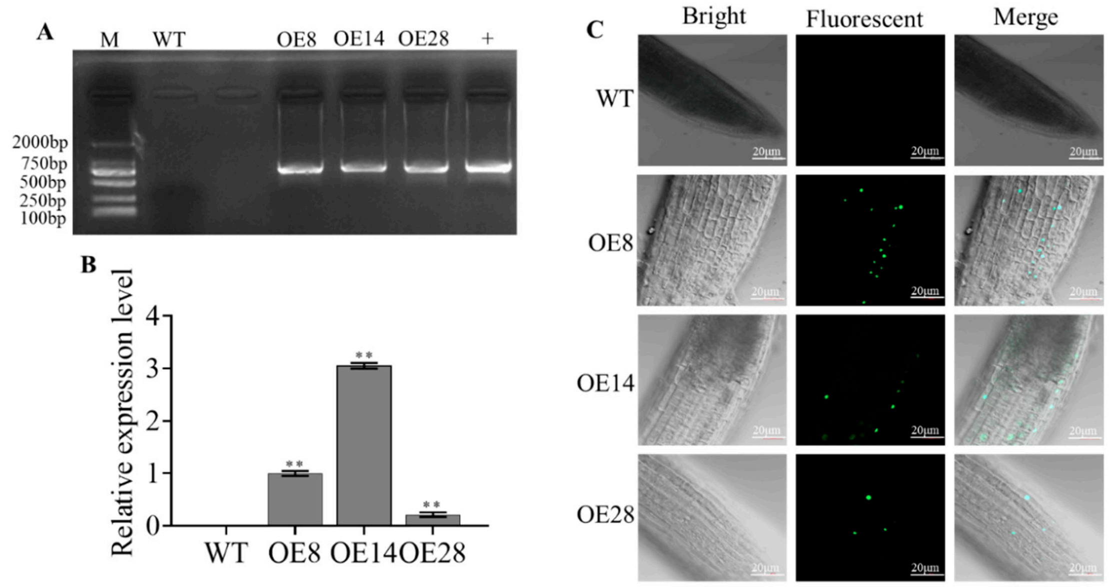

Figure S3. Identification of Arabidopsis transgenic lines overexpression of the *ZmBES1/BZR1-7* gene. A PCR dection. B qRT-PCR analysis. C GFP imaging. WT, wild type. OE8, OE14 and OE28, homozygous transgenic *Arabidopsis* lines expressing *ZmBES1/BZR1-7*. +, The *35S-ZmBES1/BZR1-7-eGFP* plasmid was used as template in PCR amplification and used as positive control.

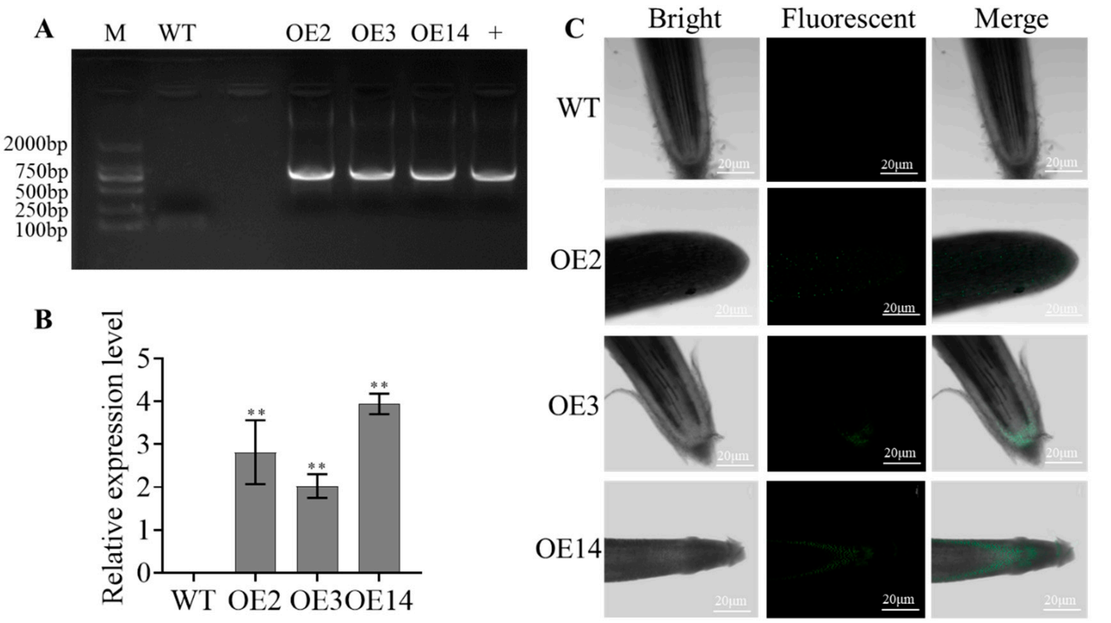

Figure S4. Identification of rice transgenic lines overexpression of the *ZmBES1/BZR1-7* gene. A PCR dection. B qRT-PCR analysis. C GFP imaging. WT, wild type. OE2, OE3 and OE14, homozygous transgenic rice lines expressing *ZmBES1/BZR1-7*. +, The *35S-ZmBES1/BZR1-7-eGFP* plasmid was used as template in PCR amplification and used as positive control.
